# Photochemical Degradation of Iron Citrate in Anoxic Viscous Films Enhanced by Redox Cascades

**DOI:** 10.1021/acsearthspacechem.4c00364

**Published:** 2025-02-25

**Authors:** Ashmi Mishra, Kevin Kilchhofer, Lucia Iezzi, Ulrich Pöschl, Peter A. Alpert, Markus Ammann, Thomas Berkemeier

**Affiliations:** † Multiphase Chemistry Department, Max Planck Institute for Chemistry, 55128 Mainz, Germany; ‡ PSI Center for Energy and Environmental Sciences, 5232 Villigen, Switzerland; ¶ Department of Environmental System Science, Institute for Atmospheric and Climate Science, ETH Zurich, 8092 Zurich, Switzerland

**Keywords:** Heterogeneous chemistry, Photochemistry, Reaction kinetics, Fenton reaction, Inverse modeling

## Abstract

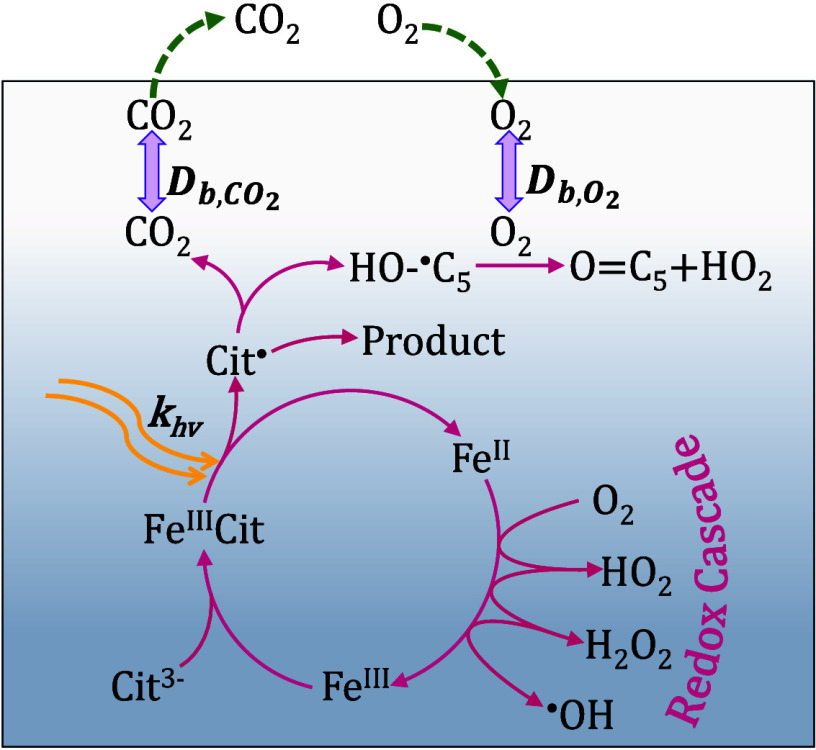

Iron contained in atmospheric aerosol particles can form complexes with organic ligands and initiate photochemical reactions that alter the composition and physicochemical properties of the particles. Depending on the temperature and humidity, organic particles exist in different phase states, which affects reactant diffusivity and chemical reaction rates. We performed coated-wall flow-tube experiments using citric acid films doped with iron as proxies for secondary organic aerosols. We quantified the CO_2_ production under UV irradiation as a function of time and relative humidity (RH) and observed a pronounced decrease of CO_2_ production with decreasing RH. The kinetic multilayer model of aerosol surface and bulk chemistry (KM-SUB) and a Monte Carlo-based global optimization method were applied to all measured data to determine the underlying effects of mass transport and chemical reactions. The model analysis revealed that after an initial rapid reaction, photooxidation becomes limited by the reoxidation of Fe^II^. Under dry conditions (RH < 65%), the reoxidation of Fe^II^ is kinetically limited by the supply of O_2_, as slow diffusion in the viscous organic matrix leads to anoxia in the interior of the film. At high humidity (RH > 85%), mass transport limitations cease, resulting in full O_2_ saturation, and photooxidation becomes limited by the chemical reaction of Fe^II^ with oxidants. Reactive oxygen species play a key role in Fe^II^ reoxidation and thus in perpetuating photooxidation chemistry. A single O_2_ molecule triggers a redox cascade from O_2_ to HO_2_, H_2_O_2_, and OH, leading to ≈3 cycles of the Fe^II^/Fe^III^ redox pair. Our model and kinetic parameters provide new insights and constraints in the interplay of microphysical properties and photochemical aging of mixed organic–inorganic aerosol particles, which may influence their effects on air quality, climate, and public health.

## Introduction

Atmospheric aerosol is a suspension of solid and liquid particles in air that plays an important role in global climate, air quality, and public health.^1,2^ Organic aerosol particles contribute to a large fraction (20 to 90%) of the total particulate mass.^3,4^ Human activities including industrial operations, brake and tire wear, as well as combustion processes can release iron into the atmosphere.^5−8^ Iron is also emitted naturally into the atmosphere in the form of mineral dust.^9^ Organic materials can act as ligands for the complexation of iron.^10^ Field studies have shown that soluble iron strongly correlates with concentrations of carboxylic acid.^11,12^ Iron carboxylates undergo photochemistry, resulting in decarboxylation and the formation of organic radicals,^13−16^ leading to the breakdown and processing of organic matter in the atmospheric aqueous phase. For such systems, the photochemical degradation of iron citrate (Fe^III^Cit) has been established as a proxy system for laboratory and modeling studies.^15,17−19^ Fe^III^ is photochemically reduced to Fe^II^ and can be reoxidized by O_2_ and reactive oxygen species (ROS), closing the photochemical cycle. Alpert et al.^18^ found that an excessive formation of ROS and organic radicals may lead to anoxic conditions as a result of the fast reaction between the radicals and oxygen and the slow diffusion of oxygen into particles exhibiting viscous phase states. Furthermore, the presence of iron will lead to the production of OH radicals through the Fenton reaction,^20^ even in the absence of light.^21^

The rate at which chemical reactions occur in organic aerosol particles is strongly dependent on their phase state,^22,23^ which varies from liquid to solid depending on composition and ambient conditions such as temperature and relative humidity (RH).^24−29^ Photochemical aging can also trigger changes in the viscosity of secondary organic aerosol (SOA) particles.^30^ Citric acid is used as a proxy for atmospheric SOA, mimicking particle hygroscopicity and viscosity.^31^ In atmospheric multiphase chemistry, kinetic multilayer models have been established as helpful tools to achieve a detailed understanding of chemical reactions and mass transport processes of particles and films.^19,32−40^

In this study, we perform coated-wall flow-tube (CWFT) experiments to quantify CO_2_ from the photolysis of Fe^III^Cit films at varying RH. To assess a possible viscosity dependence, we have carried out experiments from 30 to 85% RH. In this range, citric acid exhibits viscosities from approximately 10^2^ Pa s (30% RH) to 10^–2^ Pa s (85% RH).^18,41^ We analyze the time-resolved results of the experiments using the kinetic model KM-SUB^33^ and a Monte Carlo genetic algorithm (MCGA)^42^ for unbiased multiparameter fitting to gain mechanistic insights into the influence of microphysics on photochemical aging of mixed organic–inorganic aerosol particles.

## Methods

### Flow-Tube Coating and Experimental Setup

Coatings on the inner wall of glass flow tubes were prepared from a solution of citric acid (CitH_3_, ≥99.5%; CAS: 5949-29-1) and Fe^III^Cit tribasic monohydrate (18–20% Fe basis; CAS: 2338-05-8) purchased from Sigma-Aldrich. The dilute aqueous solutions were prepared in ultrapure water (18 M Ω cm^–1^, Milli-Q) and in a 1:10 molar ratio (CitH_3_ = 0.1 M, Fe^III^Cit = 0.01 M). The light-sensitive Fe^III^Cit solution was ensured to always be stored in the dark and was freshly prepared shortly before each experiment.

**Figure 1:**
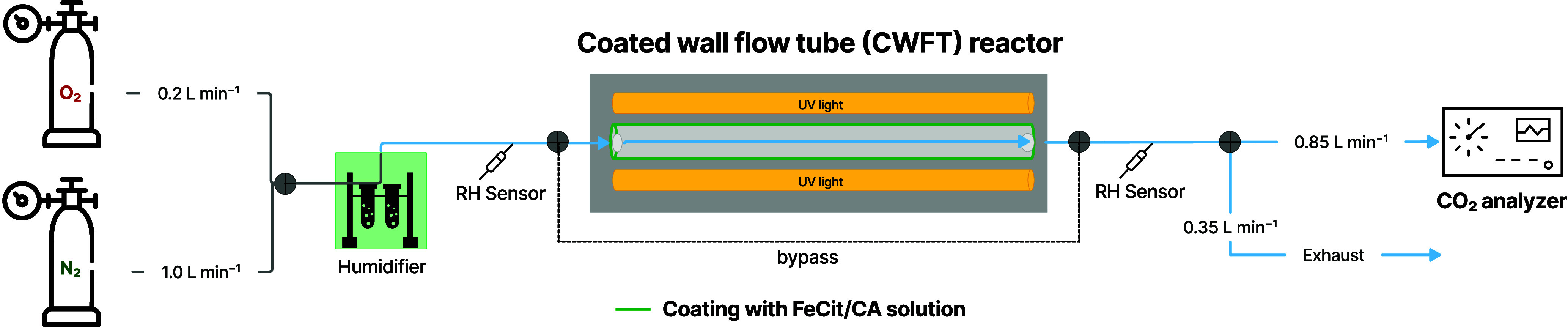
Schematic of the coated-wall flow-tube (CWFT) setup. The flow-tube was stored in a reactor casing surrounded by seven UVA lamps. The reactor casing contained silica oil to maintain *T* and RH. The geometry of the tubes was radius, *r* = 0.55 cm; length, *l* = 36 cm. The volume of the solution used for the coating was *V* = 500 μL.

The coating solution (500 μL) was pipetted into the glass tube (length: 36 cm, radius: 0.55 cm) and evenly distributed on the interior surface. We used red light while coating to not initiate excitation of Fe^III^Cit before the experiment. A moist airflow of 1.0 L min^–1^ was used to allow the film coating to settle for 40–50 min by gently tilting and turning the tube until no visible liquid remained. The RH of this airflow was set to the same RH used later in the experiment in order to pre-equilibrate the coating and to achieve a homogeneous film thickness. The glass tube was then inserted into the reactor casing containing silica oil to maintain the temperature and RH, as depicted in Figure 1, between seven UVA lamps (Phillips, 20W). There, a constant flow of 0.2 L min^–1^ O_2_ and 1.0 L min^–1^ N_2_ carrier gas with a set temperature of 21 °C under certain RH conditions was used to equilibrate the coating before each experiment. The RH was adjusted by bubbling the gas flows through a temperature-controlled water bath upstream of the CWFT reactor, which was monitored up and downstream of the CWFT. A CO_2_ analyzer (Teledyne, T200) sampled with a flow rate of 0.85 L min^–1^, and the excess flow went to exhaust.

### Experimental Procedure

Before each measurement, the background CO_2_ concentration was observed for approximately 20 min by bypassing the CWFT reactor. Upon inserting the glass tube into the reactor, the flow was directed to the CWFT, and the background concentration of CO_2_ was recorded until a steady state was reached. The film was exposed to light at two specific intervals. The irradiation periods lasted between 10 and 15 min in the first interval and between 5 and 10 min in the second interval.

Six different experiments were performed in this study, five of which were done in the presence of oxygen at 30, 35, 45, 65, and 85% RH, respectively. One experiment was performed in an oxygen-free environment at 65% RH. Table 1 provides a list of experimental parameters.

**Table 1:** Environmental Input Parameters for KM-SUB Used with the Six Experimental Data Sets: Relative Humidity (RH), Film Thickness δ_film_, Gas Phase Concentration of Oxygen [O_2_]_g_, and Bulk Phase Concentrations of Iron Citrate [Fe^III^Cit]_b_, Citric Acid [CitH_3_]_b_, and Water [H_2_O]_b_ in the Film

RH (%)	δ_film_ (cm)	[O_2_]_g_ (cm^–3^)	[Fe^III^Cit]_b_ (cm^–3^)	[CitH_3_]_b_ (cm^–3^)	[H_2_O]_b_ (cm^–3^)
30	6.12 × 10^–5^	4.74 × 10^18^	3.87 × 10^20^	3.92 × 10^21^	4.31 × 10^21^
35	6.36 × 10^–5^	4.74 × 10^18^	3.76 × 10^20^	3.81 × 10^21^	5.19 × 10^21^
45	6.80 × 10^–5^	4.74 × 10^18^	3.55 × 10^20^	3.56 × 10^21^	7.12 × 10^21^
65	8.25 × 10^–5^	4.74 × 10^18^	2.90 × 10^20^	2.94 × 10^21^	1.20 × 10^22^
85	1.24 × 10^–4^	4.74 × 10^18^	1.94 × 10^20^	1.95 × 10^21^	1.95 × 10^22^
65	6.15 × 10^–5^	0	2.90 × 10^20^	2.94 × 10^21^	1.20 × 10^22^

### Kinetic Modeling

Kinetic modeling was performed using a variant of the kinetic multilayer model of aerosol surface and bulk chemistry (KM-SUB) for planar films.^33^ The model consists of multiple compartments, including the gas phase, a sorption surface layer, and 15 bulk layers. The following processes were explicitly resolved for O_2_, CO_2_, the hydroperoxyl radical (HO_2_), and hydrogen peroxide (H_2_O_2_): gas phase diffusion, adsorption, and desorption to and from the sorption layer, bulk diffusion, as well as chemical reactions in the bulk of the film. All other reactants remain exclusively in the film bulk.

The numerical model describes the flux-based mass balance for each layer in systems of differential equations that were autogenerated using the kinetic multilayer meta model generator (KM-MEMO, Berkemeier et al., in preparation) based on the chemical mechanism and system geometry. An autogenerated Jacobian matrix is used to accelerate and increase the numerical stability of the model calculations. The KM-SUB model was modified such that the thickness of the layers increased exponentially, with the thickness of the closest layer to the surface being 0.3 nm (see the Supporting Information (SI) for details). Near the surface of the film, narrowly spaced layers ensured a smooth concentration gradient of O_2_, which allowed numerical convergence to be achieved at much lower bulk layer counts. Previous studies on similar reaction systems have introduced the Photochemical Reaction and Diffusion (PRAD) model,^19^ which solved the coupled equilibria of acid–base and complexation chemistry using instantaneous equilibration at dedicated time steps. The KM-SUB model employed here solves the coupled equilibria dynamically using explicit protonation and deprotonation reactions. We initially run the model without UV irradiation to determine the equilibrium pH. This pH remains fixed in the later calculations involving photochemistry, as the p*K*_a_ values of the products are not established in the literature. Since the Fe^III^Cit:citric acid molar ratio *M*_r_ was always 1:10 in the experiments, citric acid dominates the pH, which we find to be in the range of 1–2.

**Figure 2:**
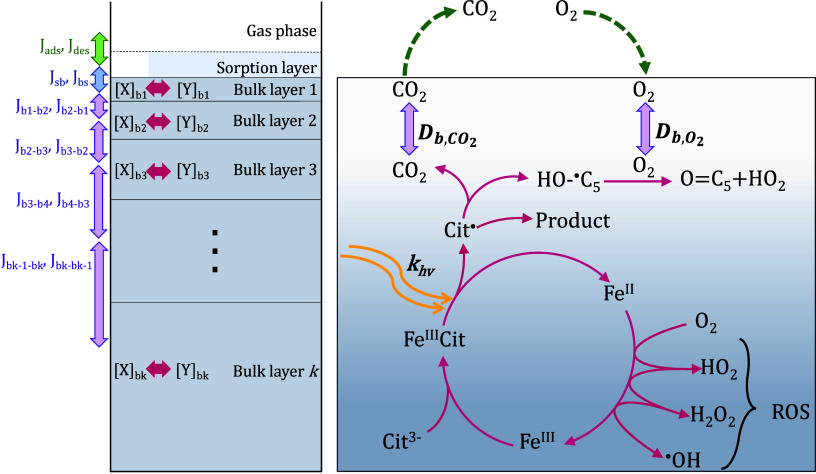
Model compartments (left) and simplified chemical mechanism (right) used in the kinetic multilayer model KM-SUB in this study. The model explicitly treats the flow reactor gas phase, a sorption surface layer, and multiple bulk layers. Transport fluxes are indicated as arrows in green (adsorption and desorption fluxes: *J*_ads_, *J*_des_), blue (mass transport between surface and bulk: *J*_sb_, *J*_bs_) and purple (mass transport within the bulk: *J*_bk,bk-1_, *J*_bk-1,bk_). The pink arrows represent the chemical reactions taking place in each bulk layer. The photochemical cycle produces reactive oxygen species (ROS) in the form of HO_2_, OH, and H_2_O_2_.

Figure 2 shows the key reactions involved in the Fe^III^Cit photochemical system to produce radicals in the presence of O_2_. Fe^III^Cit is photoactive, which after excitation forms a radical complex that quickly dissociates into Fe^II^ and an organic radical with a −C(=O)O^•^ group at the central carbon,^43,44^ Cit^•2–^, herein further referred to as Cit^•^. Cit^•^ either decarboxylates, leading to the loss of CO_2_ and production of a ketyl radical,^13,19^ or stabilizes. The branching between these determines the CO_2_ yield. Note that as the reactions of Cit^•^ are not well established in the literature, we fit a first-order loss rate to minimize the number of fitting parameters. The ketyl radical can react with another Fe^III^Cit complex to yield Fe^II^ citrate (Fe^II^CitH) and ketone.^13^ Reactive oxygen species (ROS: H_2_O_2_, HO_2_, O_2_^–^, OH) are generated when O_2_ taken up by the film is reduced, e.g., through the ketyl radical, or through Fe^II^ being reoxidized to Fe^III^, closing the photochemical cycle. Cit^•^ is also formed through the reaction of the hydroxyl radical (OH) with citrate. Table 2 and Tables S1 and S2 provide a list of all model input parameters used in this study. In the fitting of diffusion coefficients, we prescribed a monotonous increase with relative humidity.

**Table 2:** KM-SUB Kinetic Input Parameters^a^

parameter	range	description	reference
*D*_b,O2_	1.0 × 10^–13^–1.0 × 10^–7^ (cm^2^ s^–1^)	bulk diffusion coefficient of O_2_	this study
*D*_b,CO2_	1.0 × 10^–13^–1.0 × 10^–7^ (cm^2^ s^–1^)	bulk diffusion coefficient of CO_2_	this study
*D*_b,Fe^III^Cit_ 30% RH	[1.0 × 10^–15^] (cm^2^ s^–1^)	bulk diffusion coefficient of Fe^III^Cit	Dou et al.^19^
*D*_b,Fe^III^Cit_ 35% RH	[3.0 × 10^–15^] (cm^2^ s^–1^)	bulk diffusion coefficient of Fe^III^Cit	Dou et al.^19^
*D*_b,Fe^III^Cit_ 45% RH	[1.0 × 10^–13^] (cm^2^ s^–1^)	bulk diffusion coefficient of Fe^III^Cit	Dou et al.^19^
*D*_b,Fe^III^Cit_ 65% RH	[2.0 × 10^–12^] (cm^2^ s^–1^)	bulk diffusion coefficient of Fe^III^Cit	Dou et al.^19^
*D*_b,Fe^III^Cit_ 85% RH	[5.0 × 10^–12^] (cm^2^ s^–1^)	bulk diffusion coefficient of Fe^III^Cit	Dou et al.^19^
*D*_b,H2O_ 30% RH	[3.0 × 10^–9^] (cm^2^ s^–1^)	bulk diffusion coefficient of H_2_O	Dou et al.^19^
*D*_b,H2O_ 35% RH	[5.0 × 10^–9^] (cm^2^ s^–1^)	bulk diffusion coefficient of H_2_O	Dou et al.^19^
*D*_b,H2O_ 45% RH	[1.0 × 10^–7^] (cm^2^ s^–1^)	bulk diffusion coefficient of H_2_O	Dou et al.^19^
*D*_b,H2O_ 65% RH	[2.0 × 10^–6^] (cm^2^ s^–1^)	bulk diffusion coefficient of H_2_O	Dou et al.^19^
*D*_b,H2O_ 85% RH	[4.0 × 10^–6^] (cm^2^ s^–1^)	bulk diffusion coefficient of H_2_O	Dou et al.^19^
*H*_cp,O2_	[1.32 × 10^–6^] (mol cm^–3^ atm^–1^)	Henry’s law solubility coefficient of O_2_	Sander^45^
*H*_cp,CO2_	[3.34 × 10^–5^] (mol cm^–3^ atm^–1^)	Henry’s law solubility coefficient of CO_2_	Sander^45^
α_s,0,CO2_, α_s,0,O2_	[1]	surface accommodation coefficient of CO_2_ and O_2_	-
τ_D,O2_	[1.4 × 10^–11^] (s^–1^)	desorption lifetime of O_2_	Knopf et al.^46^
τ_D,CO2_	[4.5 × 10^–9^] (s^–1^)	desorption lifetime of CO_2_	Knopf et al.^46^
*a*_Γ,CO2_	1–10	CO_2_ analyzer response curve, shape parameter	this study
*b*_Γ,CO2_	1–10	CO_2_ analyzer response curve, scale parameter	this study

a The respective lower and upper boundaries indicate the initial constraints of the fit ensemble. Values that were fixed during the fitting procedure are indicated using square brackets.

### Global Optimization

The kinetic model KM-SUB was fitted to experimental observations using a global optimization algorithm (Monte Carlo genetic algorithm, MCGA).^42^ Model input parameters are inferred by simultaneously fitting the kinetic model to all of the available experimental data. MCGA is comprised of two steps. The first step consists of performing a Monte Carlo search by randomly sampling parameters from a range of predefined boundaries. The globally best-fitting parameter sets are then fed into the starting population of GA, in which they are optimized by processes mimicking survival, recombination, and mutation in evolutionary biology. The optimization was stopped when the population was homogeneous, and therefore, the increase in correlation with the experimental data ceased. Ideally, global optimization leads to a unique fit, i.e., a parameter set that is invariant when repeating the fitting process. In practice, however, multiphase chemical systems are often underdetermined, as they contain too many or nonorthogonal parameters or there are not enough data to achieve a unique fit.^37,42^ In such instances, finding kinetic parameter sets that best fit the data is less beneficial. Thus, in this study, we identified an ensemble of adequately fitting parameter sets and collectively analyzed the kinetic model solutions that corresponded to those parameter sets.

## Results and Discussion

### CO_2_ Production

**Figure 3:**
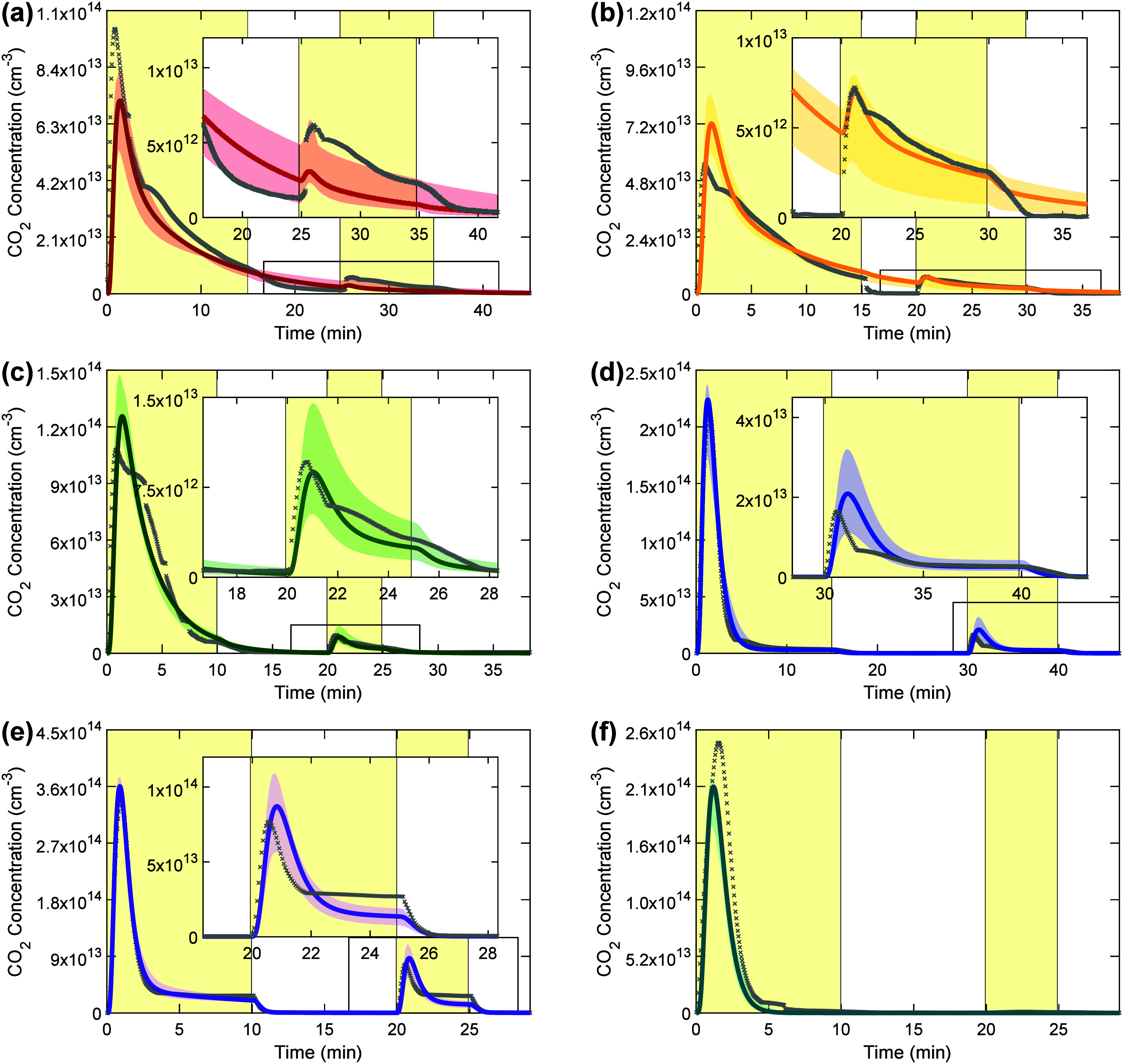
CO_2_ production from the photolysis of iron citrate films in the coated-wall flow-tube experiments (gray markers) and the corresponding KM-SUB modeling results (dark colored lines) at different RH (30% (a), 35% (b), 45% (c), 65% (d), 85% (e), and 65% in the absence of O_2_ (f)). The colored solid lines represent the globally best-fitting kinetic parameter set, and the colored shadings indicate the variability within the fit ensemble of *N* = 55 fits. The light yellow shaded regions represent irradiation periods.

Figure 3 shows the gas phase number concentration of CO_2_ produced in the coated-wall flow-tube (CWFT) experiments and compares the measurement with the KM-SUB model results. The experimental data are shown using gray markers, while the model results are depicted with solid lines and colored shadings. Using the MCGA, we generated an ensemble (*N* = 55) of model parameter sets that exhibit a satisfactory correlation to the experimental data. The lines depict the best-fitting model result, while the shadings denote the range of model results in the fit ensemble. The light yellow shaded regions show periods in which irradiation with UV light occurred. There is a stark difference in the intensity of the CO_2_ signal between irradiation and dark periods. During the irradiation periods, the CO_2_ concentrations increase sharply, peaking within seconds of the light being turned on. We see an increase in the peak concentrations with increasing RH, with the experiment at 85% RH (panel e) showing ∼4 times higher CO_2_ compared to the lowest RH experiment (30%, panel a). At lower RH (30–45%, panels a–c), the CO_2_ signal decreases continuously after the initial peak, while at higher RH (65–85%, panels d and e), the signal eventually reaches a plateau after minutes of irradiation, which points toward a steady state of CO_2_ production and evaporation. The model ascribes the sharp peak in the CO_2_ concentration to rapid photolysis and depletion of Fe^III^Cit. Continued CO_2_ production is limited by the availability of Fe^III^Cit for photolytic degradation and, to a lesser extent, limited by the formation of hydroxyl radicals (OH) that can react with citrate.

We note that while all Fe^III^Cit photolyzes within minutes, the area under the initial CO_2_ peak is smaller than the total amount of Fe^III^Cit in the system. Hence, we assume that not all photolyzed Fe^III^Cit leads to the production of CO_2_. We find a CO_2_ yield, defined as the ratio of the production of CO_2_ and the photolysis loss of Fe^III^Cit, of around 15%, irrespective of the experimental conditions (Figure S1). We find that a higher CO_2_ yield cannot be compensated by lowering the Fe^III^Cit photolysis rate in the model, as this leads to a broadening of the initial CO_2_ peak (Figure S2). We also note that the CO_2_ peaks in the experimental data show a shoulder feature that is not captured by the model. This is seen in all but the highest humidity experiments and may be related to the presence of other photoactive species such as carboxylate complexes of reaction products with iron or a dinuclear iron citrate complex.^43^

During dark periods, the CO_2_ signals tend toward zero. The decay of the CO_2_ signal during dark periods is governed by the diffusivity of the CO_2_ in the model. The CO_2_ peak from the second irradiation period is generally much smaller than the initial CO_2_ peak, which indicates that the film has not returned to its original state after 5–10 min of darkness. In the model, this is due to the incomplete reoxidation of Fe^II^ to Fe^III^. The height of the second peak delivers important information about the O_2_ diffusivity and Fe^II^ reoxidation rate. Generally, at higher RH, O_2_ diffuses more quickly into the film, leading to faster Fe^II^ reoxidation. This is corroborated by experiments in the absence of O_2_ (panel f), in which the film’s ability to produce CO_2_ is not recovered in the dark. Furthermore, OH cannot be formed via ROS cycling in the absence of O_2_, meaning there is no formation of Cit^•^ through this reaction channel. In the following, we discuss the RH-dependent mass transport limitations of CO_2_ and O_2_ in greater detail.

### Regeneration of Fe^III^Cit by Oxidation Reactions

**Figure 4:**
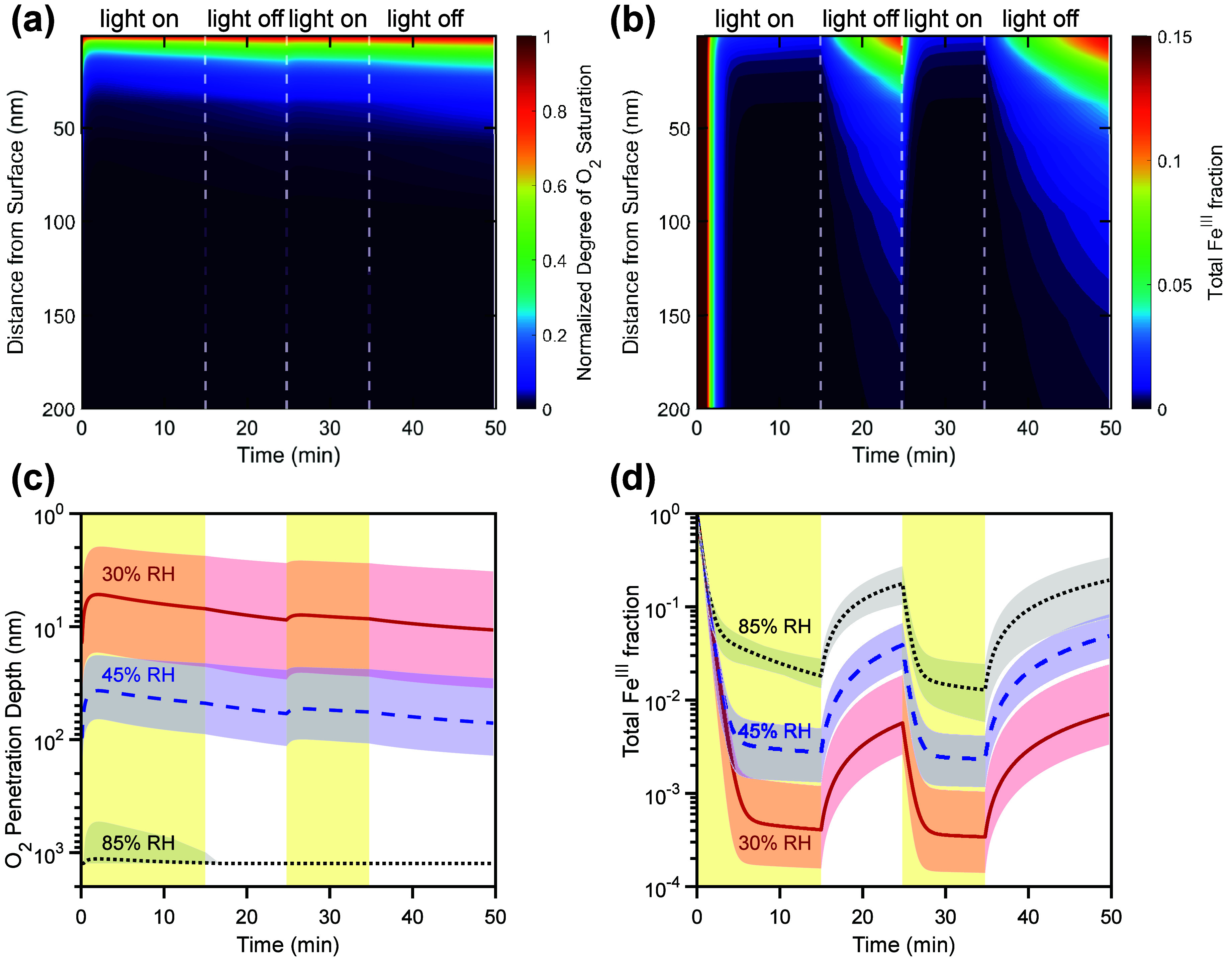
Depth profiles of the O_2_ concentration (a) and Fe^III^ fraction (b) in a film as a function of time at 30% RH. The *y*-axis indicates the distance from the film surface. (c) Penetration depth of O_2_, i.e., the position in the film at which the O_2_ saturation reaches 0.5. (d) Model results for the total Fe^III^ fraction as a function of time. In panels (c) and (d), the solid lines represents the mean model result obtained from the ensemble solution, while the shaded areas indicate the ensemble variability. The light yellow shadings represent irradiation periods. For ease of comparison, the irradiation periods were simulated for all humidities as they occurred in the 30% RH experiment. Note that panels (a) and (b) only depict the top 200 nm of the film.

Panels a and b of Figure 4 show the simulated depth profiles of the normalized degree of oxygen saturation (*S*_O_2__ = [O_2_]_bulk_/[O_2_]_bulk,sat_]) and the fraction of Fe^III^ to total iron ([Fe^III^]/([Fe^II^]+[Fe^III^])) in the topmost 200 nm of the CWFT film, respectively, for the lowest humidity experiment (30% RH). We find that high saturation of O_2_ only occurs very close to the surface. The iron concentration in the film (∼500 mM) is much larger than the saturation concentration of O_2_ (∼0.25 mM), causing anoxia upon irradiation with light. This indicates that O_2_ diffusion is slow compared to reactive consumption, leading to a reacto-diffusive kinetic regime.^47^ The reacto-diffusive length^48^ and penetration depth of O_2_ (Figure 4c) are near-constant over the course of the experiment. We define the penetration depth as the point at which *S*_O_2__ reaches 0.5, which occurs at ∼5–10 nm for the experiment at 30% RH. Accordingly, the reoxidation of Fe^II^ back to Fe^III^ also occurs close to the surface. Notably, the shapes of the depth profiles of oxygen saturation and Fe^III^ fraction differ visually due to the different time evolutions of their sink terms. While the O_2_ is removed at a near-constant rate, Fe^III^ only recovers during dark periods of the experiment in the absence of its strong photochemical sink. Note that recomplexation of Fe^3+^ with citrate is not a limiting step in the chemical mechanism, and the steady-state concentration of Fe^III^ is much higher than that of the free Fe^3+^ ion in the model. Full depth profiles of oxygen saturation and Fe^III^ fraction for all experiments are provided in the Supporting Information (Figures S3 and S4).

Note that O_2_ is not the only oxidant of Fe^II^ in this system. In the model, the dominant Fe^II^ species is Fe^II^CitH. In fact, H_2_O_2_ and HO_2_ are the species that contribute most to the oxidation of Fe^II^ in the model simulations (Figure S5). However, O_2_ starts the chain of ROS production, and its reactive uptake to the film perpetuates photooxidation of citric acid. In the model, O_2_ reacts with a ketyl radical and Fe^II^ to produce HO_2_. We find Fe^II^ to be the main sink for HO_2_ and H_2_O_2_, and hence, every O_2_ molecule that diffuses into the film triggers a redox cascade from O_2_ to HO_2_, H_2_O_2_, and OH. We find that HO_2_ and H_2_O_2_ react predominantly with Fe^II^, yielding at least three equivalents of Fe^II^ oxidation per O_2_ taken up into the film. Evaporation of HO_2_ and H_2_O_2_ from the film constituted a minor loss channel in all model calculations. In the case of the very reactive OH radical, the largest sink is citrate rather than Fe^II^, and therefore, it does not contribute significantly to Fe^II^ oxidation. We note, however, that the oxidation products of the reaction between citrate with OH (i.e., alkyl and peroxy radicals) may decarboxylate themselves, undergo radical–radical recombination, or oxidize Fe^II^.^49^ The model currently does not consider the downstream organic radical chemistry toward further degradation of citric acid.

### Humidity Dependence of Reactant Concentration Profiles

**Figure 5:**
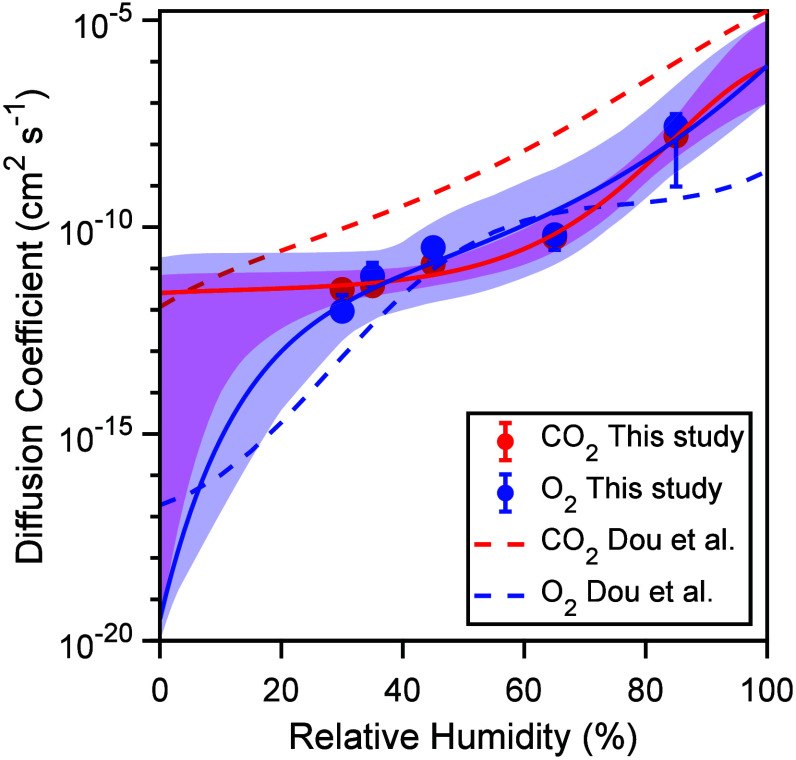
Bulk diffusion coefficients of O_2_ (blue markers) and CO_2_ (red markers) as functions of humidity. The shaded blue and red areas represent ensembles of fits to a Vignes-type equation (eq 2), with the blue and red lines showing the best fits for O_2_ and CO_2_, respectively.

Figure 4c,d compares the O_2_ penetration depth and Fe^III^ fraction for different humidities (35, 45, and 85% RH). Upon increasing the RH, increased bulk diffusion coefficients (Figure 5) lead to a larger reaction zone of O_2_ with Fe^II^. At 85% RH, O_2_ and ROS reach deep into the bulk of the film, and we observe the largest recovery of Fe^III^ in the simulation of this experiment, accordingly. However, despite full saturation of O_2_, the Fe^III^ fraction recovers to at most ∼0.25 after initial irradiation, which means that the reaction with Fe^II^ must limit O_2_ uptake, indicating a kinetic regime limited by chemical reaction. This is in line with Alpert et al.^18^ and Dou et al.,^19^ who showed that even after almost 2 h in the dark, Fe^III^Cit particles do not reach a Fe^III^ fraction of 1. This incomplete reoxidation affects the extent of CO_2_ production during the second irradiation period, resulting in the peak concentration of the second irradiation being smaller than the peak concentration of CO_2_ during the initial irradiation period at all humidities (Figure 3). Nonetheless, faster diffusion of O_2_ at high humidity leads to higher CO_2_ production in the second irradiation period, as observed in the experiments.

Due to the relatively short chemical lifetime of HO_2_, we find its concentration profile to track the penetration depth of O_2_ closely (Figure S6). As H_2_O_2_ is less reactive than HO_2_, it is more evenly distributed in the film and found to be well-mixed at 65% RH and above (Figure S7).

### Humidity Dependence of Diffusion Coefficients

Figure 5 shows the humidity dependence of the bulk diffusion coefficients of CO_2_ and O_2_ as determined by MCGA. The closed markers represent the mean diffusion coefficients from the ensemble of fitting parameter sets. We find that both diffusion coefficients increase with RH. The diffusion coefficients of both O_2_ and CO_2_ are overall similar, as one would expect from their molecular size. The diffusion coefficients are fitted using a Vignes-type equation as a function of the molar fraction of organics (*x*_org_), as introduced in Lienhard et al.:^31^
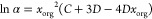
1
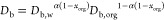
2

The best-fitting Vignes-type parametrizations are shown as solid lines. For uncertainty estimation, we sampled the parameters *C*, *D*, *D*_b,org_, and *D*_b,w_ 100 000 times within predetermined fitting boundaries (Table S3) following a Monte Carlo Markov Chain (Metropolis–Hastings algorithm) and selected an ensemble of fits based on two acceptance criteria: a monotonous increase of the diffusion coefficients with RH and a residual less than twice that of the best fit. The ensemble of Vignes-type fits shows a range of behaviors encompassing a double S-shaped curve as seen in Berkemeier et al.,^35^ as well as a single S-shaped curve as seen for citric acid in Lienhard et al.^31^ Without experimental data at humidities <30% RH, we find a larger uncertainty in this humidity range and therefore cannot distinguish between both behaviors; however, we find that the best fit for CO_2_ exhibits a single S-shape, while the best fit for O_2_ shows a double S-shape. The diffusion coefficients of O_2_ and CO_2_ inferred in this study follow a similar trend as in the study by Dou et al.^14^ on the same reaction system. Dou et al.^19^ found a higher diffusivity of CO_2_ compared to the CO_2_ in this study. Note that, in Dou et al.,^14^ CO_2_ diffusivity was inferred based on particle mass loss, which is also affected by water uptake into the particle, while this study uses time profiles of CO_2_ gas phase concentration. As CO_2_ is formed only during irradiation, switching between light and dark periods allows the disentangling of chemical and mass transport kinetics of CO_2_ production in the film. Diffusion coefficients are kept fixed during the simulations for simplicity. However, product formation could affect the diffusion coefficients. The diffusion may increase as smaller organic molecules are formed^19^ or may decrease as dimers are produced.^15^

## Atmospheric Relevance

**Figure 6:**
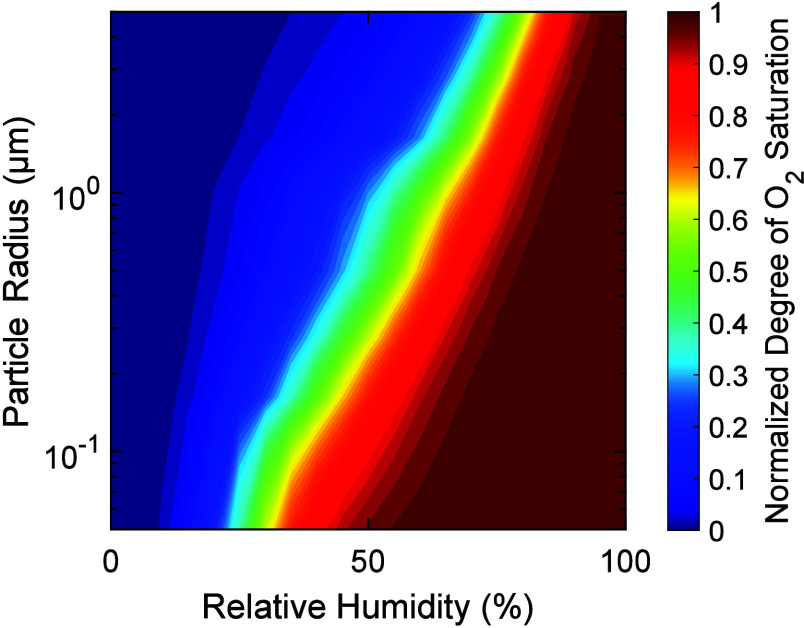
Degree of oxygen saturation (*S*_O_2__) as a function of particle size and relative humidity. The photon flux was set to solar irradiation at the Earth’s surface at a zenith angle of 0°. The Fe^III^Cit concentration was 0.41 M following Khaled et al.,^50^ and citric acid was added to maintain a Fe^III^Cit to organics mole ratio of 1:10.

To assess the impact of iron carboxylate photochemistry on SOA particles in the atmosphere, we extrapolate the kinetic model from laboratory to atmospheric conditions by changing the geometry from thin films to spherical particles, reducing the Fe^III^ concentrations to 0.41 M,^50^ which is a concentration representative of iron-containing aerosol particles, and using a photon flux according to solar irradiation at the Earth’s surface at a zenith angle of 0°. Figure 6 shows the normalized degree of oxygen saturation (*S*_O_2__) in the particles after 12 h of solar irradiation as a function of particle size and RH. Particle size and humidity were kept constant during the simulations. Figure 6 uses the best-fitting Vignes-type parametrization (Figure 5) for the diffusion coefficients. We find that at low humidities, particles become fully anoxic, irrespective of the particle size, while at medium RH, *S*_O_2__ depends strongly on the particle size. At high humidities (>70%), oxygen remains fully saturated in the simulations, irrespective of particle size. Thus, for dilute aerosols and cloud water, we expect a well-mixed reaction system in which oxidative processing is limited by the chemical reaction of Fe^II^ with O_2_ and ROS. We note that the oxygen concentration shown here is the volume-weighted, average bulk concentration, and anoxia will be more pronounced in the interior of particles. Panels a and b of Figure S8 are generated using the upper and lower limits of the diffusion coefficients in Figure 5, respectively. Using the upper estimate of the diffusion coefficients (Figure S8a), we see a particle size dependence in *S*_O_2__ at both low and medium humidities: *S*_O_2__ becomes as low as ∼0.05 for large particles. Using the lower estimate of diffusion coefficients (Figure S8b), we find a similar behavior to that of the best fit: at low humidities particles become fully anoxic, irrespective of the particle size, while at medium RH, *S*_O_2__ depends strongly on particle size. In both diffusivity limits, at high humidities, oxygen is fully saturated in the simulations for all particle sizes.

## Conclusions

In this study, we performed detailed kinetic laboratory experiments and kinetic modeling on the iron citrate heterogeneous photooxidation system, which serves as an important proxy for the aging of transition-metal-containing organic aerosol particles, and thus the breakdown of organic matter, in the atmosphere.^51^ Iron carboxylate photochemistry is considered a dominant sink for carboxylic acids compared to oxidation with radicals such as OH.^51^ Our model simulations corroborate these findings and show that CO_2_ production through iron citrate photolysis dominates over CO_2_ production through oxidation with the OH radical under the conditions studied here. The time-resolved and humidity-dependent data from the coated-wall flow-tube system, using an experimental protocol that includes light-on and light-off periods, allowed the kinetic model to disentangle the effects of phase state and chemical reaction. The combined experiments and model calculations demonstrate that relative humidity and therefore the phase state can strongly affect the rate of chemical aging of organic aerosol particles. We show the effects of relative humidity on the diffusion of O_2_ and CO_2_ and find that compared to previous studies, the diffusion coefficients of these two molecules are very similar.

Using the kinetic model, we can distinguish two distinct kinetic regimes of photooxidation. At low humidities, O_2_ only penetrates the film close to the surface due to the fast chemical consumption and kinetic transport limitation in the viscous film. At high humidities, we find that O_2_ can fully penetrate the bulk of the film and that the reactions with Fe^II^ are limiting O_2_ uptake. We find that the absence of oxygen stops further decarboxylation from occurring, while the presence of O_2_ results in a cascade of reactive oxygen species (ROS) formation that perpetuates redox cycling and thus the photooxidation reaction, which is limited by the availability of reduced Fe. We note that other photosensitizers are also known to initiate free radical chemistry by reacting with O_2_ to produce HO_2_, even in the absence of transition metals.^52,53^ Using the physical and chemical parameters derived from the kinetic model, we extrapolate the model from the laboratory to atmospheric conditions. We find that at low and intermediate RH, the presence of anoxic conditions will strongly depend on particle size.

Uncertainties remain in the chemical mechanism with respect to minor and later generation reaction products. We find that only ≈15% of Fe^III^Cit photolysis leads to the formation of CO_2_, which is determined by the chemical fate of Cit^•^. The downstream chemistry of Cit^•^ has not been well studied and is simplified in the model to a first-order loss rate. Detailed analysis of the fate of Cit^•^ could help in the understanding of the lowered CO_2_ yield. Furthermore, the reaction of CitH_3_ with OH yields an alkyl radical, which may decarboxylate, or in the presence of O_2_ forms peroxy radicals. The downstream chemistry of organic radicals is currently not considered in the model. This could mean that under high humidity conditions, our model may underestimate sinks of O_2_.

Future experimental studies are needed to reduce the model uncertainty. We suggest that future studies include simultaneous experimental data of other observables from the system, such as measurements of reactive oxygen species (e.g., gas phase HO_2_), or a more detailed chemical analysis of oxidation products, such as volatile organic decomposition products in the gas phase (e.g., acetic acid) or nonvolatile radical–radical recombination products in the condensed phase, to help constrain the model and chemical mechanism of the reaction system further. Future studies should also investigate the effects of temperature since it affects both the phase state and chemical reaction rates.

## Supporting Information Available

CO_2_ yield as a function of humidity, comparison of model results with 100% CO_2_ yield, depth profiles of O_2_ and total Fe^III^ at different humidities, relative sinks of Fe^II^ as a function of time and humidity, depth profiles of HO_2_ and H_2_O_2_ at different humidities, *S*_O_2__ as a function of particle size and relative humidity at two different diffusion coefficient extremes, acid–base equilibria, chemical reactions, input parameters for the Vignes-type equation, and additional method details (PDF)
