# Supplementary material for: Photochemical Degradation of Iron Citrate in Anoxic Viscous Films Enhanced by Redox Cascades
Source: ACS Earth Space Chem. 2025 Feb 25;9(3):689–98. doi: 10.1021/acsearthspacechem.4c00364 (PMC11931546; doi:10.1021/acsearthspacechem.4c00364)
Supplement: Supplementary file 1 — sp4c00364_si_001.pdf [file sp4c00364_si_001.pdf]

Supporting Information for: Photochemical degradation of iron citrate in anoxic viscous films enhanced by redox cascades

Ashmi Mishra[1], Kevin Kilchhofer[2,3], Lucia Iezzi[2,3], Ulrich Pöschl[1], Peter A. Alpert[2], Markus Ammann[2], and Thomas Berkemeier[1]

[1]Multiphase Chemistry Department, Max Planck Institute for Chemistry, 55128 Mainz, Germany
[2]PSI Center for Energy and Environmental Sciences, 5232 PSI Villigen, Switzerland
[3]Department of Environmental System Science, Institute for Atmospheric and Climate Science, ETH Zurich, 8092 Zurich, Switzerland

Correspondence: Thomas Berkemeier (t.berkemeier@mpic.de)

Fig. S1 CO_2 yield as a function of humidity

Fig. S2 Comparison of model results with 100% CO_2 yield

Fig. S3 Depth profile of O_2 at different humidities

Fig. S4 Depth profile of total Fe^{III} at different humidities

Fig. S5 Relative sinks of Fe^{II} as a fucntion of time and humidity

Fig. S6 Depth profile of HO_2 at different humidities

Fig. S7 Depth profile of H_2O_2 at different humidities

Fig. S8 Degree of oxygen saturation (S_{O_2}) as a function of particle size and relative humidity

Tab. S1 Acid-base equilibria

Tab. S2 Chemical reactions

Tab. S3 Input parameters for Vignes-type equation

Sect. S1 Layer sizing in the kinetic model

Sect. S2 Water activity parameterization

Sect. S3 Film Thickness calculation

1 Supplementary Figures

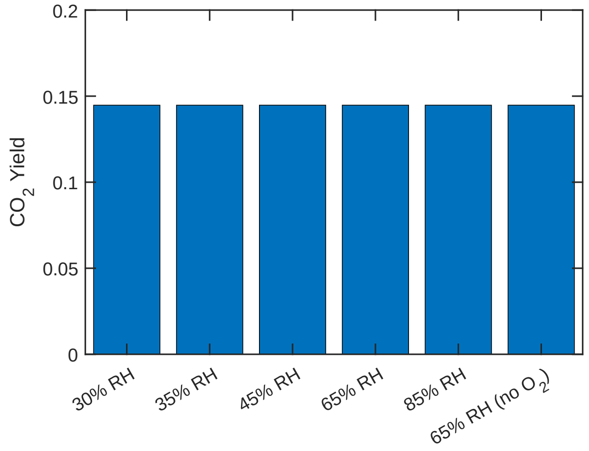

Figure S1. CO_2 yield as a function of humidity. The CO_2 yield is defined as the number of CO_2 molecules produced per loss of Fe^{III}Cit

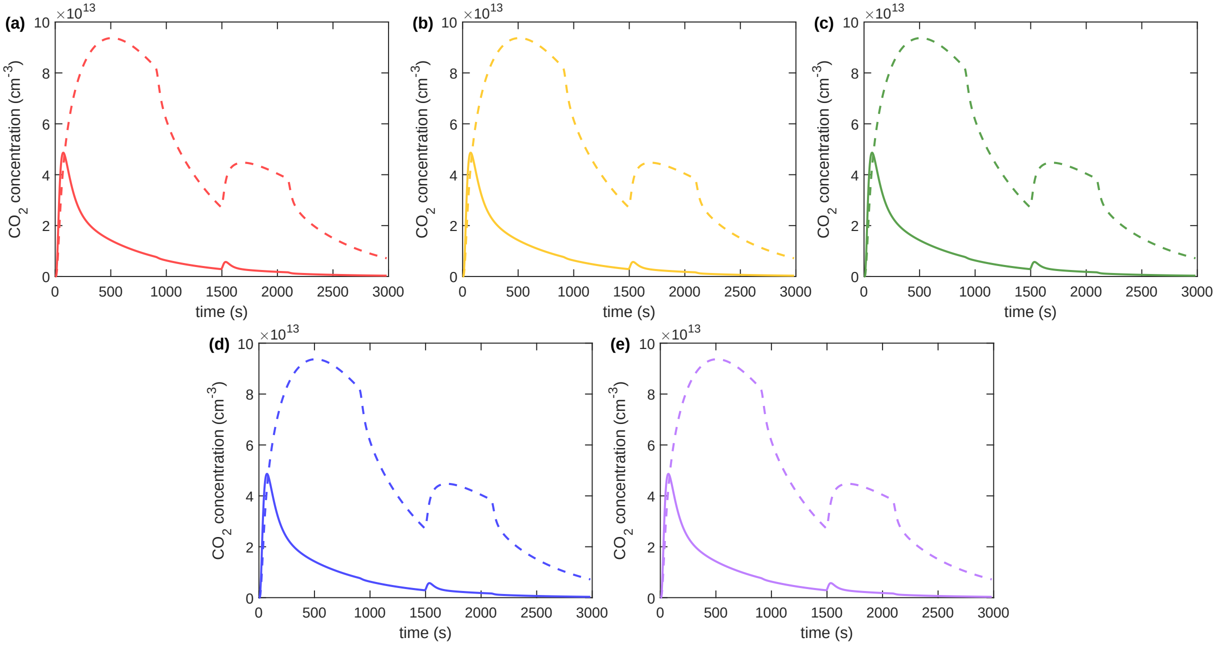

Figure S2. CO_2 concentration with best model fit parameters (solid lines) and a comparison with a model result assuming 100% CO_2 yield and a photolysis rate that is a factor of 15 lower than in the best model fit (dashed lines) to partially compensate for the higher CO_2 yield. Results are displayed as a function of time at different relative humidities (30 % RH (a), 35 % RH (b), 45 % RH (c), 65 % RH (d), 85 % RH (e)).

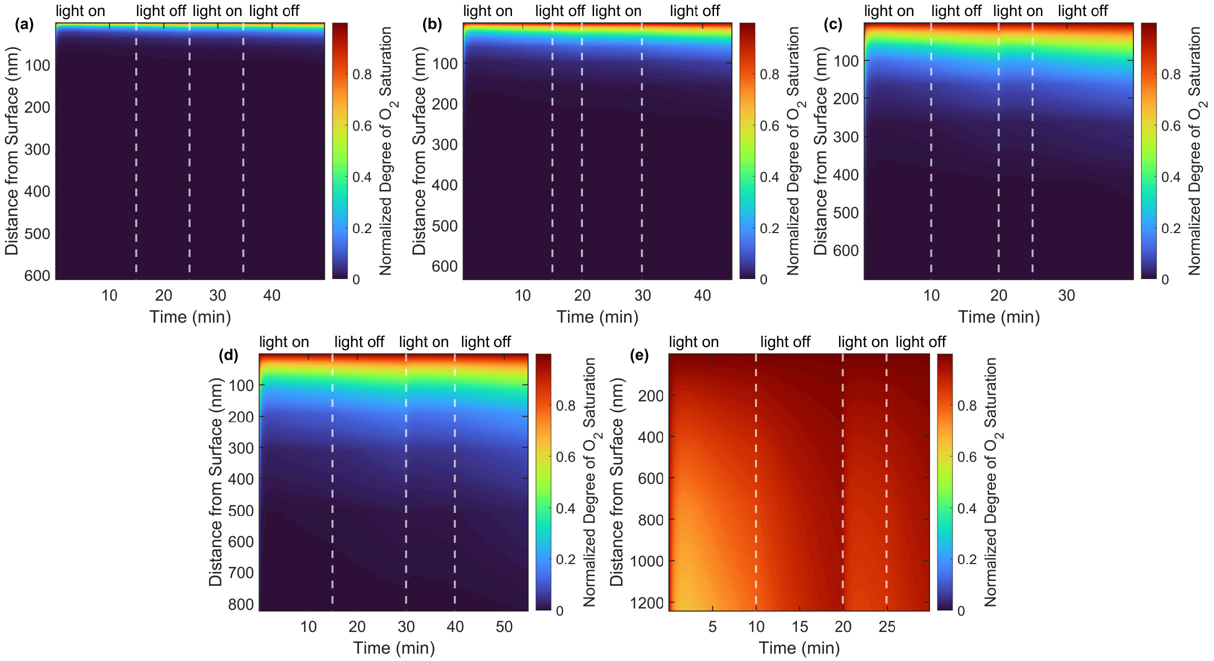

Figure S3. Model results for the depth profile of O_2 in the films as a function of time at different relative humidities (30 % RH (a), 35 % RH (b), 45 % RH (c), 65 % RH (d), 85 % RH (e)). The y-axis indicates the distance from the film surface.

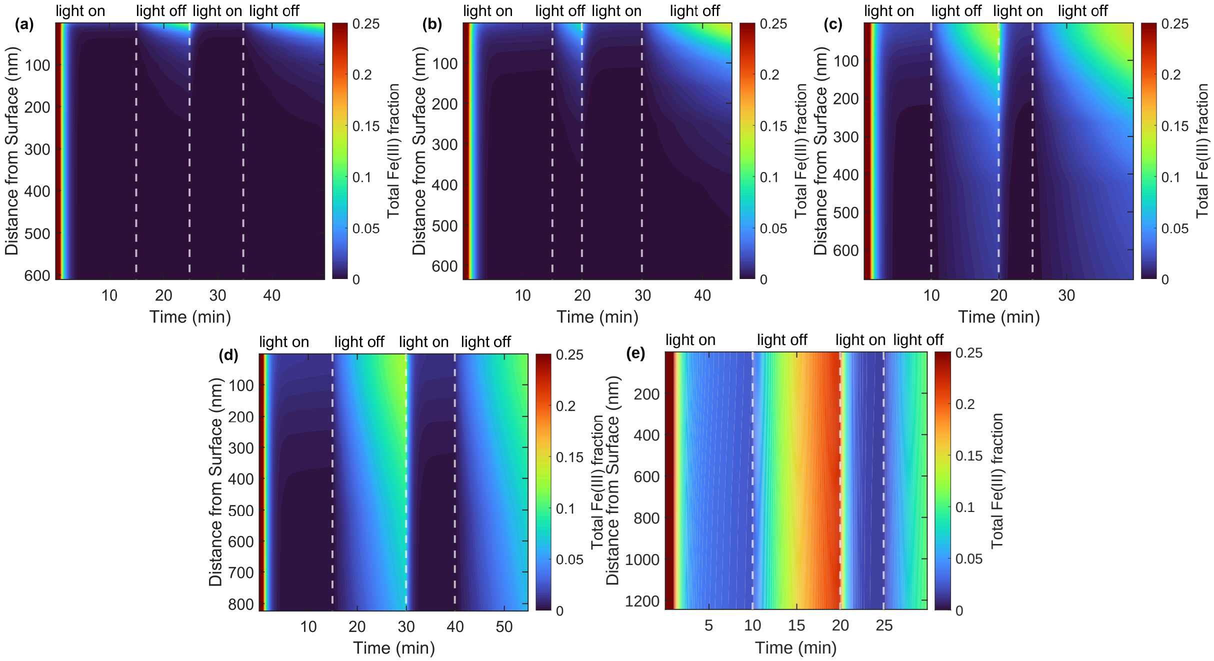

Figure S4. Model results for the depth profile of Fe^{III} fraction in the films as a function of time at different relative humidities (30 % RH (a), 35 % RH (b), 45 % RH (c), 65 % RH (d), 85 % RH (e)). The y-axis indicates the distance from the film surface.

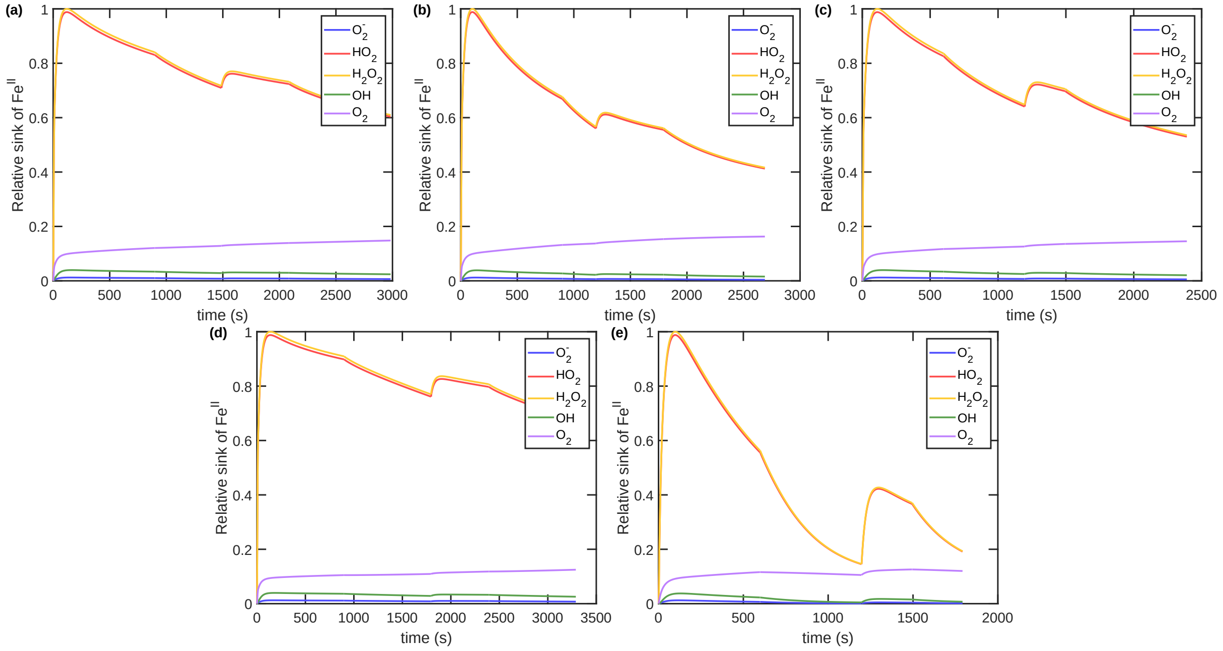

Figure S5. Relative Fe^{II}CitH sinks as a function of time at different relative humidities (30 % RH (a), 35 % RH (b), 45 % RH (c), 65 % RH (d), 85 % RH (e)).

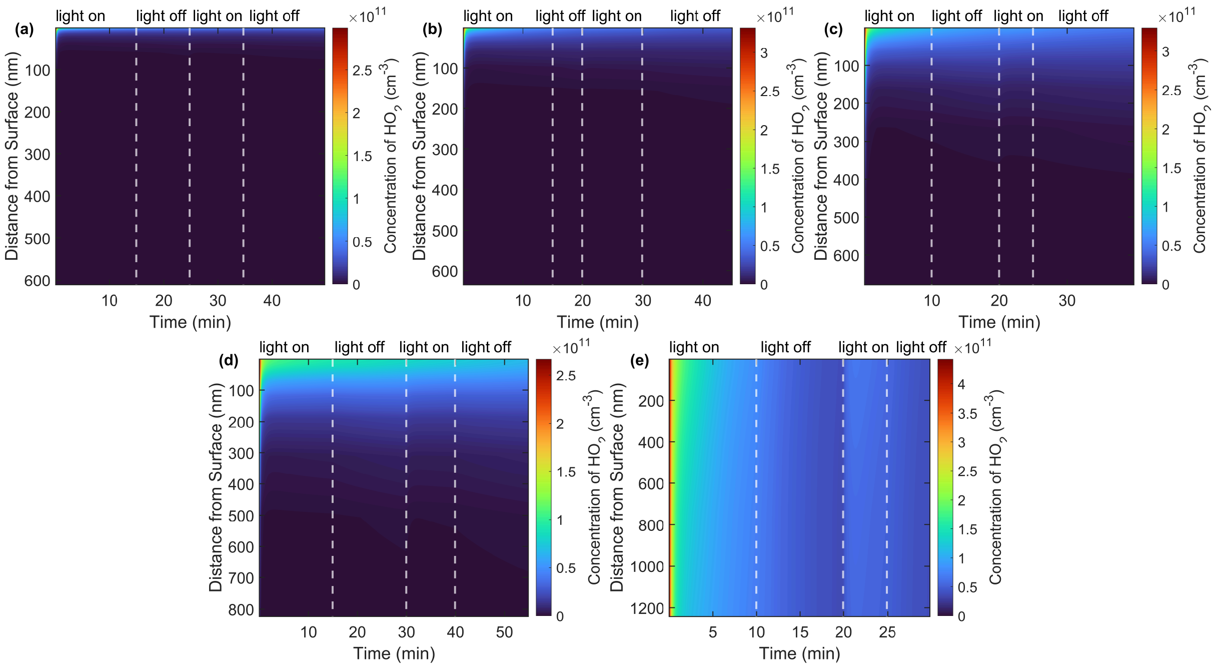

Figure S6. Model results for the depth profile of HO_2 in the films as a function of time at different relative humidities (30 % RH (a), 35 % RH (b), 45 % RH (c), 65 % RH (d), 85 % RH (e)). The y-axis indicates the distance from the film surface.

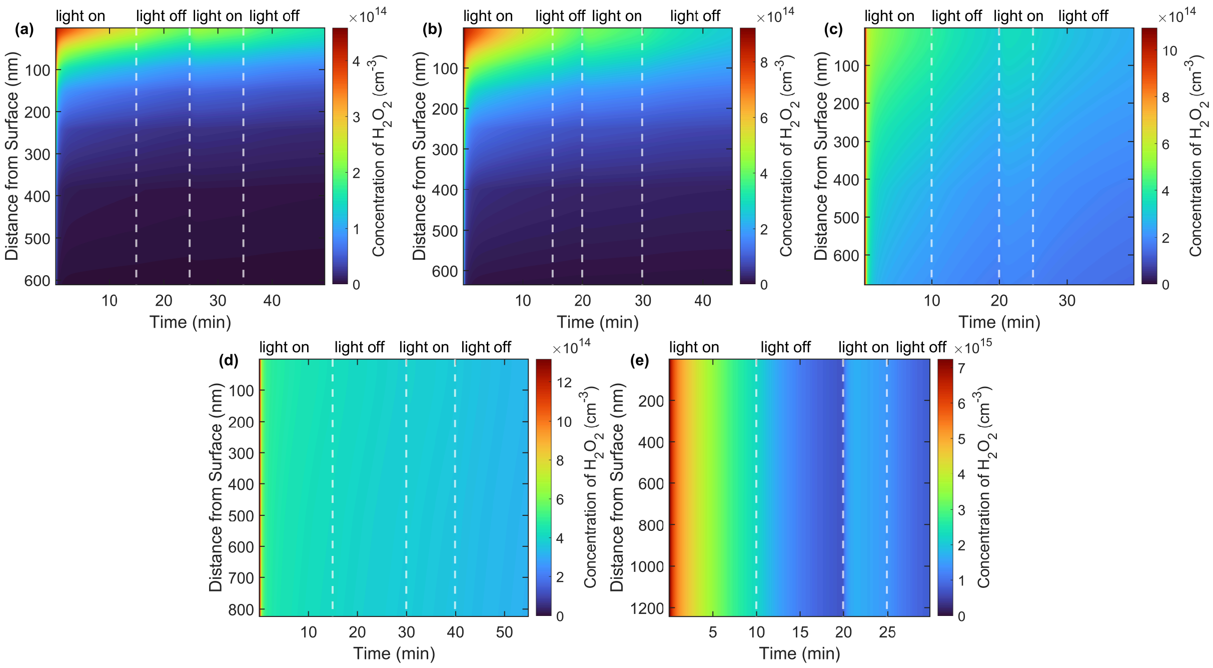

Figure S7. Model results for the depth profile of H_2O_2 in the films as a function of time at different relative humidities (30 % RH (a), 35 % RH (b), 45 % RH (c), 65 % RH (d), 85 % RH (e)). The y-axis indicates the distance from the film surface.

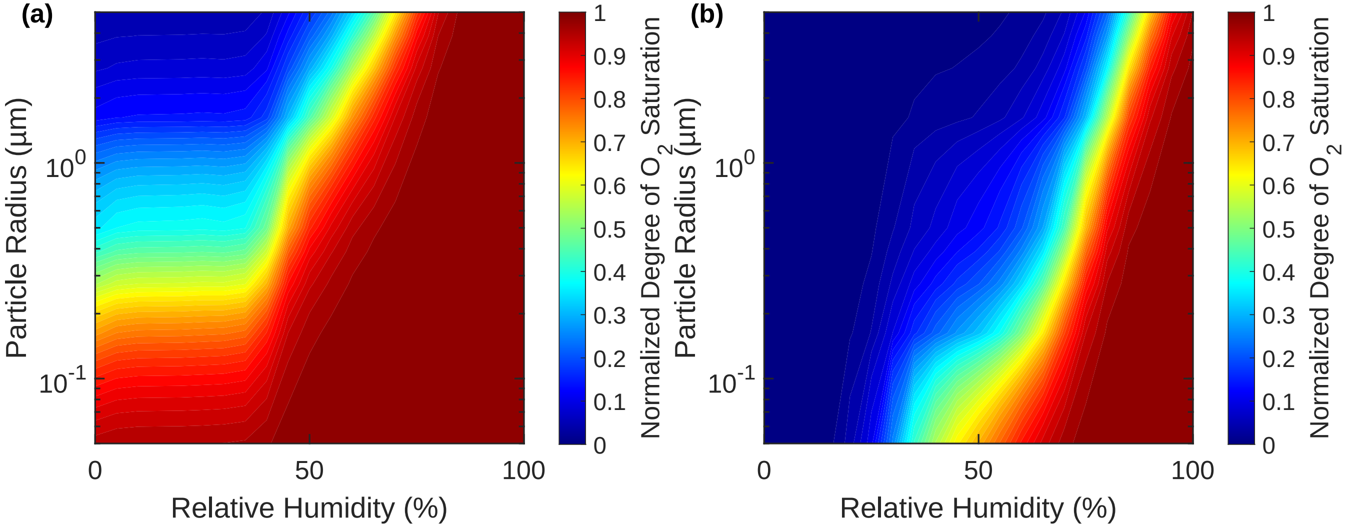

Figure S8. Normalized degree of oxygen saturation (S_{O_2} = $[O_2]_{bulk}/[O_2]_{bulk,sat}$) as a function of particle size and relative humidity at two different extremes of diffusion coefficients (panel a: $D_{b,CO2}$: 2.00 × 10^{-20} – 1.00 × 10^{-7} cm^2 s^{-1}, $D_{b,O2}$: 1.00 × 10^{-20} – 1.00 x 10^{-7} cm^2 s^{-1}, panel b: $D_{b,CO2}$: 6.90 × 10^{-12} – 9.80 × 10^{-6} cm^2 s^{-1}, $D_{b,O2}$: 1.90 × 10^{-11} – 1.00 × 10^{-5} cm^2 s^{-1}). The photon flux was set to solar irradiation at the Earth's surface at a zenith angle of 0°. The Fe^{III}Cit concentration was 0.41 M following Khaled et al. (2022) and citric acid was added to maintain a Fe^{III}Cit to organics mole ratio of 1:10.

2 Supplementary Tables

Table S1. Chemical reaction equilibria used in this study. In the KM-SUB model, the forward reaction rate is set to 1×10^{8} M^{-1} s^{-1} and the backward reaction rate is derived from the equilibrium constant.

Number	Equilibrium reaction	Equilibrium constant	Reference
1	$OH^{-} + H^{+} \rightleftharpoons H_2O$	1.80×10^{-16} M	
2	$CitH_2{}^{-} + H^{+} \rightleftharpoons CitH_3$	1.33×10^{3} M^{-1}	Martell and Smith (1982)
3	$CitH^{2-} + H^{+} \rightleftharpoons CitH_2{}^{-}$	5.88×10^{4} M^{-1}	Martell and Smith (1982)
4	$Cit^{3-} + H^{+} \rightleftharpoons CitH^{2-}$	2.50×10^{6} M^{-1}	Martell and Smith (1982)
5	$Fe^{3+} + Cit^{3-} \rightleftharpoons Fe^{III}Cit$	1.58×10^{13} M^{-1}	Dou et al. (2021)
6	$[Fe^{III}CitOH]^{-} + H^{+} \rightleftharpoons Fe^{3+} + Cit^{3-} + H_2O$	1.20×10^{-8} M	Dou et al. (2021)
7	$Fe^{3+} + CitH^{2-} \rightleftharpoons [Fe^{III}CitH]^{+}$	2.51×10^{7} M^{-1}	Dou et al. (2021)
8	$Fe^{2+} + CitH^{2-} \rightleftharpoons Fe^{II}CitH$	1.94×10^{10} M^{-1}	Dou et al. (2021)
9	$[Fe^{III}OH]^{2+} + H^{+} \rightleftharpoons Fe^{3+} + H_2O$	4.57×10^{-3} M	Smith and Martell (1976)
10	$Fe^{2+} + O{=}C(CH_2COO)_2{}^{2-} \rightleftharpoons Fe^{II}[O{=}C(CH_2COO)_2]$	2.00×10^{-3} M^{-1}	Dou et al. (2021)
11	$2\,H^{+} + O{=}C(CH_2COO)_2{}^{2-} \rightleftharpoons O{=}C(CH_2COOH)_2$	1.50×10^{6} M^{-2}	Dou et al. (2021)
12	$2\,H^{+} + OH{-}C(CH_2COO)_2{}^{2-} \rightleftharpoons OH{-}C(CH_2COOH)_2$	1.50×10^{6} M^{-2}	Dou et al. (2021)
13	$O_2{}^{-} + H^{+} \rightleftharpoons HO_2$	6.30×10^{4} M^{-1}	Bielski et al. (1985)
14	$Fe^{3+} + CitH_3 \rightleftharpoons Fe^{III}Cit + 3\,H^{+}$	8.06×10^{-2} M^{-2}	Inferred from 2, 3, 4, 5
15	$[Fe^{III}OH]^{2+} + CitH^{2-} \rightleftharpoons Fe^{III}Cit + H_2O$	1.38×10^{9}	Inferred from 4, 5, 9
16	$[Fe^{III}CitOH]^{-} + CitH_3 \rightleftharpoons Fe^{III}Cit + CitH_3 + OH^{-}$	1.42×10^{2} M	Inferred from 2, 5, 9
17	$[Fe^{III}CitH]^{+} + CitH_2{}^{-} \rightleftharpoons Fe^{III}Cit + CitH_3$	3.35×10^{2}	Inferred from 2, 4, 5, 7
18	$[Fe^{III}CitH]^{+} + Cit^{3-} \rightleftharpoons Fe^{III}Cit + CitH^{2-}$	6.29×10^{5}	Inferred from 5, 7
19	$[Fe^{III}CitOH]^{-} + CitH_2{}^{-} \rightleftharpoons Fe^{III}Cit + CitH^{2-} + H_2O$	3.22 M	Inferred from 3, 5, 6
20	$[Fe^{III}CitOH]^{-} + CitH^{2-} \rightleftharpoons Fe^{III}Cit + Cit^{3-} + H_2O$	7.57×10^{-2} M	Inferred from 4, 5, 6
21	$[Fe^{III}OH]^{2+} + CitH_2{}^{-} \rightleftharpoons Fe^{III}Cit + H^{+} + H_2O$	2.35×10^{4} M	Inferred from 3, 4, 5, 9
22	$[Fe^{III}OH]^{2+} + CitH_3 \rightleftharpoons Fe^{III}Cit + 2\,H^{+} + H_2O$	1.76×10^{1} M^{2}	Inferred from 2, 3, 4, 5, 9
23	$[Fe^{III}OH]^{2+} + Cit^{3-} \rightleftharpoons Fe^{III}Cit + OH^{-}$	3.46×10^{1}	Inferred from 1, 5, 9
24	$[Fe^{III}CitH]^{+} + CitH^{2-} \rightleftharpoons Fe^{III}Cit + CitH_2{}^{-}$	1.48×10^{4}	Inferred from 3, 4, 5, 7
25	$Fe^{3+} + CitH_2{}^{-} \rightleftharpoons Fe^{III}Cit + 2\,H^{+}$	1.07×10^{2} M	Inferred from 3, 4, 5

Table S2. Chemical reactions included in the KM-SUB model. The respective lower and upper boundaries indicate the initial constraints of the fit ensemble. Values that were fixed during the fitting procedure are indicated using square brackets. Species on top of the arrow in the chemical reaction refer to those that partake in the chemical reaction but that are not used to determine the reaction order.

Reaction	Range	Reference
$Fe^{III}Cit + h\nu \rightarrow Fe^{2+} + Cit^{\bullet}$	2.65×10^{-2} - 1.06×10^{-1} s^{-1}	This study
$Cit^{\bullet} \rightarrow OH-C(CH_2COO)_2{}^{2-} + CO_2$	[1.00×10^{9} s^{-1}]	Pozdnyakov et al. (2014)
$OH-C(CH_2COO)_2{}^{2-} + Fe^{III}Cit \rightarrow Fe^{2+} + O=C(CH_2COO)_2{}^{2-} + CitH^{2-}$	8.20×10^{-23} - 8.20×10^{-19} cm^3 s^{-1}	This study
$Cit^{\bullet} \rightarrow Cit^{3-}$	1.00×10^{8} - 1.00×10^{10} s^{-1}	This study
$HO_2 + HO_2 \rightarrow H_2O_2 + O_2$	[3.45×10^{-12} cm^3 s^{-1}]	Ervens et al. (2003)
$HO_2 + O_2{}^{-} \xrightarrow{H_2O} H_2O_2 + O_2 + OH^{-}$	[1.70×10^{-13} cm^3 s^{-1}]	Ervens et al. (2003)
$O_2{}^{-} + O_2{}^{-} \xrightarrow{2H+} H_2O_2$	[3.82×10^{-16} cm^3 s^{-1}]	Ervens et al. (2003)
$H_2O_2 + OH \rightarrow HO_2 + H_2O$	[5.50×10^{-14} cm^3 s^{-1}]	Ervens et al. (2003)
$OH + OH \rightarrow H_2O_2$	[8.60×10^{-12} cm^3 s^{-1}]	Ervens et al. (2003)
$OH + O_2{}^{-} \rightarrow O_2 + OH^{-}$	[1.30×10^{-11} cm^3 s^{-1}]	Ervens et al. (2003)
$OH + HO_2 \rightarrow O_2 + H_2O$	[1.20×10^{-11} cm^3 s^{-1}]	Ervens et al. (2003)
$H_2O_2 + HO_2 \rightarrow OH + O_2 + H_2O$	[4.98×10^{-21} cm^3 s^{-1}]	Ervens et al. (2003)
$Fe^{2+} + O_2 \rightarrow Fe^{3+} + O_2{}^{-}$	8.31×10^{-23} - 4.16×10^{-21} cm^3 s^{-1}	This study
$Fe^{2+} + O_2{}^{-} \xrightarrow{2H+} H_2O_2 + Fe^{3+}$	[1.66×10^{-14} cm^3 s^{-1}]	Rush and Bielski (1985)
$Fe^{2+} + HO_2 \xrightarrow{H+} H_2O_2 + Fe^{3+}$	[1.99×10^{-15} cm^3 s^{-1}]	Rush and Bielski (1985)
$Fe^{2+} + H_2O_2 \rightarrow Fe^{3+} + OH + OH^{-}$	[1.26×10^{-19} cm^3 s^{-1}]	Walling (1975)
$Fe^{2+} + OH \rightarrow Fe^{III}OH$	[7.14×10^{-13} cm^3 s^{-1}]	Christensen and Sehested (1981)
$Fe^{II}[O=C(CH_2COO)_2] + O_2 \rightarrow O_2{}^{-} + Fe^{3+} + O=C(CH_2COO)_2{}^{2-}$	8.31×10^{-23} - 4.16×10^{-21} cm^3 s^{-1}	This study
$Fe^{II}[O=C(CH_2COO)_2] + O_2{}^{-} \xrightarrow{2H+} H_2O_2 + Fe^{3+} + O=C(CH_2COO)_2{}^{2-}$	[1.66×10^{-14} cm^3 s^{-1}]	Rush and Bielski (1985)
$Fe^{II}[O=C(CH_2COO)_2] + HO_2 \xrightarrow{H+} H_2O_2 + Fe^{3+} + O=C(CH_2COO)_2{}^{2-}$	[1.99×10^{-15} cm^3 s^{-1}]	Rush and Bielski (1985)
$Fe^{II}[O=C(CH_2COO)_2] + H_2O_2 \rightarrow Fe^{3+} + OH + OH^{-} + O=C(CH_2COO)_2{}^{2-}$	[1.26×10^{-19} cm^3 s^{-1}]	Walling (1975)
$Fe^{II}[O=C(CH_2COO)_2] + OH \rightarrow Fe^{III}OH + O=C(CH_2COO)_2{}^{2-}$	[7.14×10^{-13} cm^3 s^{-1}]	Christensen and Sehested (1981)
$Fe^{II}CitH + O_2 \rightarrow Fe^{III}Cit + HO_2$	8.31×10^{-23} - 4.16×10^{-21} cm^3 s^{-1}	This study
$Fe^{II}CitH + HO_2 \rightarrow H_2O_2 + Fe^{III}Cit$	[1.99×10^{-15} cm^3 s^{-1}]	Rush and Bielski (1985)
$Fe^{II}CitH + H_2O_2 \rightarrow Fe^{III}Cit + OH + H_2O$	[1.26×10^{-19} cm^3 s^{-1}]	Walling (1975)
$Fe^{II}CitH + OH \rightarrow Fe^{III}Cit + H_2O$	[7.14×10^{-13} cm^3 s^{-1}]	Christensen and Sehested (1981)
$OH-C(CH_2COO)_2{}^{2-} + O_2 \rightarrow O=C(CH_2COO)_2{}^{2-} + O_2{}^{-} + H^{+}$	[1.66×10^{-15} cm^3 s^{-1}]	Dou et al. (2021)
$Organics + OH \rightarrow (1-\alpha)product + (\alpha)Cit^{\bullet}$	[1.66×10^{-12} cm^3 s^{-1}]	This study

Table S3. Input parameters used in the correction factor α (Eq. 1) and Vignes-type equation (Eq. 2) and their optimization boundaries.

	C	D	$D_{\mathrm{b,org}}$	$D_{\mathrm{b,w}}$
O_2	-5 – 5	-5 – 5	1.0×10^{-20} – 1.0×10^{-11} cm^2 s^{-1}	1.0×10^{-7} – 1.0×10^{-5} cm^2 s^{-1}
CO_2	-5 – 5	-5 – 5	1.0×10^{-20} – 1.0×10^{-11} cm^2 s^{-1}	1.0×10^{-7} – 1.0×10^{-5} cm^2 s^{-1}

3 Supplementary Text

3.1 Layer sizing in the kinetic model

The thickness (d) of each bulk layer in the chemical model was altered such that each layer is not equally spaced but rather that the layers closer to the surface are thinner while those in the deep bulk are thicker.

$$d(i) = 3.46 \times 10^{-8} f^{(i-1)}; \tag{1}$$

where i is the layer number and f is a factor required such that the sum of d is equal to the total film thickness.

3.2 Water activity parameterization

The water activity (a_w) was parameterized using the mass fraction of citric acid, following Lienhard et al. (2012):

$$a_w = \frac{1 - mfs}{(1 + q \times mfs + r \times mfs^2)} \tag{2}$$

where,

$$q = -3.16761 + 0.01939T - 4.02725 \times 10^{-5} T^2 \tag{3}$$

$$r = 6.59108 - 0.05294T + 1.06028 \times 10^{-4} T^2 \tag{4}$$

Dou et al. (2021) showed that the water activity of an aqueous 1 M iron citrate solution corresponds to the water activity of a 0.81 M aqueous citric acid solution. Hence, following Dou et al. (2021), we further parameterize $\mathrm{Fe^{III}Cit}$ using a correction factor of 0.81, yielding the apparent citrate moles to be:

$$nCit = n\mathrm{CitH_3} + 0.81 \times nFe^{\mathrm{III}}Cit \tag{5}$$

We consider that the citric acid and $Fe^{III}Cit$ solution is a "pure" citric acid solution which has water activity a_w. We can then use equation 2 to calculate $mfs_{\mathrm{CitH_3}}$, and the correction factor 0.81 to calculate the moles of "citric acid" in the film. By using the molecular weight of citric acid, we get the mass fraction of the solutes in equation 2.

3.3 Film Thickness Calculation

The film thickness in the flow-tube is described by the volume of the film (V_{film}) divided by the inner surface of the flow-tube (A_{ft}):

$$h_{\mathrm{film}} = \frac{V_{\mathrm{film}}}{A_{\mathrm{ft}}} \tag{6}$$

where,

$$A_{\mathrm{ft}} = 2\pi \cdot r_{\mathrm{ft}} \cdot l_{\mathrm{ft}}, \tag{7}$$

and the volume with the mass of the film:

$$V_{\mathrm{film}} = \frac{m_{\mathrm{film}}}{\rho} \tag{8}$$

The mass of the film refers to the mass fractions of the solutes in the film, as calculated above in section 3.2. The density of the solution is calculated as a weighted mean of the density of the solutes.

$$\rho = \frac{n_{\mathrm{CitH_3}} \cdot \rho_{\mathrm{CitH_3}} + n_{Fe^{\mathrm{III}}Cit} \cdot \rho_{Fe^{\mathrm{III}}Cit}}{n_{tot}} \tag{9}$$

The density of citric acid is taken from Lienhard et al. (2012). $\rho_{Fe^{\mathrm{III}}Cit}$ is directly proportional to $\rho_{\mathrm{CitH_3}}$, where the proportional factor is the ratio between the densities of the pure solids (ρ°). Thus, equation 9 becomes:

$$\rho = \rho_{\mathrm{CitH_3}} \cdot \left(\frac{n_{\mathrm{CitH_3}} + n_{Fe^{\mathrm{III}}Cit} \cdot \frac{\rho^\circ_{Fe^{\mathrm{III}}Cit}}{\rho^\circ_{\mathrm{CitH_3}}}}{n_{\mathrm{CitH_3}} + n_{Fe^{\mathrm{III}}Cit}}\right) \tag{10}$$

References

Bielski, B. H. J., Cabelli, D. E., Arudi, R. L., and Ross, A. B.: Reactivity of HO2/O-2 Radicals in Aqueous Solution, J. Phys. Chem. Ref. Data, 14, 1041–1100, https://doi.org/10.1063/1.555739, 1985.

Christensen, H. and Sehested, K.: Pulse radiolysis at high temperatures and high pressures, Radiat. Phys. Chem., 18, 723–731, https://doi.org/10.1016/0146-5724(81)90195-3, 1981.

Dou, J., Alpert, P. A., Corral Arroyo, P., Luo, B., Schneider, F., Xto, J., Huthwelker, T., Borca, C. N., Henzler, K. D., Raabe, J., Watts, B., Herrmann, H., Peter, T., Ammann, M., and Krieger, U. K.: Photochemical degradation of iron(III) citrate/citric acid aerosol quantified with the combination of three complementary experimental techniques and a kinetic process model, Atmos. Chem. Phys., 21, 315–338, https://doi.org/10.5194/acp-21-315-2021, 2021.

Ervens, B., George, C., Williams, J. E., Buxton, G. V., Salmon, G. A., Bydder, M., Wilkinson, F., Dentener, F., Mirabel, P., Wolke, R., and Herrmann, H.: CAPRAM 2.4 (MODAC mechanism): An extended and condensed tropospheric aqueous phase mechanism and its application, J. Geophys. Res., 108, https://doi.org/10.1029/2002JD002202, 2003.

Khaled, A., Zhang, M., and Ervens, B.: The number fraction of iron-containing particles affects OH, HO_2 and H_2O_2 budgets in the atmospheric aqueous phase, Atmos. Chem. Phys., 22, 1989–2009, https://doi.org/10.5194/acp-22-1989-2022, 2022.

Lienhard, D. M., Bones, D. L., Zuend, A., Krieger, U. K., Reid, J. P., and Peter, T.: Measurements of Thermodynamic and Optical Properties of Selected Aqueous Organic and Organic–Inorganic Mixtures of Atmospheric Relevance, J. Phys. Chem. A, 116, 9954–9968, https://doi.org/10.1021/jp3055872, 2012.

Martell, A. E. and Smith, R. M.: Critical Stability Constants: First Supplement, Boston, MA, oCLC: 851794557, 1982.

Pozdnyakov, I. P., Melnikov, A. A., Tkachenko, N., Chekalin, S. V., Lemmetyinen, H., and Plyusnin, V. F.: Ultrafast photophysical processes for Fe(iii)-carboxylates, Dalton Trans., 43, 17 590–17 595, https://doi.org/10.1039/C4DT01419G, 2014.

Rush, J. D. and Bielski, B. H. J.: Pulse radiolytic studies of the reaction of perhydroxyl/superoxide O2- with iron(II)/iron(III) ions. The reactivity of HO2/O2- with ferric ions and its implication on the occurrence of the Haber-Weiss reaction, J. Phys. Chem., 89, 5062–5066, https://doi.org/10.1021/j100269a035, 1985.

Smith, R. M. and Martell, A. E.: Critical Stability Constants: Inorganic Complexes, Cham, oCLC: 1159214048, 1976.

Walling, C.: Fenton's reagent revisited, Acc. Chem. Res., 8, 125–131, https://doi.org/10.1021/ar50088a003, 1975.
